# Dose Response and Prediction Characteristics of a Methylation Sensitive Digital PCR Assay for Cigarette Consumption in Adults

**DOI:** 10.3389/fgene.2018.00137

**Published:** 2018-04-24

**Authors:** Robert Philibert, Meesha Dogan, Amanda Noel, Shelly Miller, Brianna Krukow, Emma Papworth, Joseph Cowley, Jeffrey D. Long, Steven R. H. Beach, Donald W. Black

**Affiliations:** ^1^Behavioral Diagnostics LLC, Coralville, IA, United States; ^2^Department of Psychiatry, University of Iowa, Iowa City, IA, United States; ^3^Cardio Diagnostics, Coralville, IA, United States; ^4^Center for Alcohol and Drug Services, Davenport, IA, United States; ^5^Center for Family Studies at the University of Georgia, Athens, GA, United States

**Keywords:** smoking, epigenetics, DNA methylation, AHRR, cg05575921, digital PCR, diagnostics

## Abstract

The tobacco use disorders are the largest preventable cause of morbidity and mortality in the world. A substantial barrier to the development of better intervention and screening measures is the lack of clinically employable biomarkers to detect the existence and extent of tobacco consumption. In prior work, we and others have shown that array based assessment of DNA methylation status at cg05575921 is a sensitive and quantitative method for assessing cigarette consumption. Unfortunately, in general, arrays are not practical clinical tools. Herein, we detail the prediction performance metrics and dose dependency of a clinically implementable droplet digital PCR (ddPCR) assay for cigarette consumption in adults. First, we demonstrate that measurements of cg05575921 as determined by Illumina array and ddPCR are highly correlated (*R*^2^ = 0.98, *n* = 92). Second, using clinical data and biomaterial from 177 subjects ranging from 18 to 78 years of age, we show that the Receiver Operating Characteristic (ROC) area under the curve (AUC) for classifying smoking status using methylation status at cg05575921 is 0.99. Finally, we conduct modeling analyses of cigarette consumption over discrete time periods to show that methylation status is best correlated with mean cigarette consumption over the past year (*R*^2^ = 0.5) and that demethylation at cg05575921 is dose dependent with a demethylation (delta beta) of 1% being equivalent to 1.2 cigarettes per day. But we do not find a relationship between Fagerstrom score and DNA methylation. We conclude that ddPCR assessment of cg05575921 methylation is an accurate method for assessing the presence and extent of cigarette consumption in adult subjects. We suggest that skillful clinical implementation of this approach alone or in combination with other assessment methods could lead to substantial reduction of cigarette consumption related morbidity and mortality.

## Introduction

The tobacco use disorders are the largest cause of morbidity and mortality in the United States (Centers for Disease Control and Prevention, 2015). Despite the extensive use of preventive measures and the development of modestly effective pharmacotherapies, the rate of smoking in United States adults remains relatively fixed at ~18% (Centers for Disease Control and Prevention, 2011). Although there are a number of reasons for the persistence of this deadly addiction, one major barrier is the lack of clinically employable biomarkers to assess the presence and extent of cigarette smoking.

The main method to screen for smoking is self-report. In epidemiologic settings, self-report is reasonably reliable (Benowitz et al., 2009). However, in health care settings where consequences could conceivably occur, self-report is considerably less reliable. For example, at the health care payor level, although ~18% of Iowa adults smoke, only 7% of the 1.4 million adults covered by Wellmark Blue Cross and Blue Shield reported that they smoke (Leys, 2014). Along with driving up the healthcare costs for those who do not smoke, this misreporting also deprives the insurance company a potential opportunity to provide and/or incentivize smoking cessation interventions. At the point of contact level, self-report, in particular for high risk patients, can be equally unreliable. For example, up to 39% of pregnant women who are positive for cotinine deny smoking while 36% of lung transplant recipients are positive for cotinine yet deny smoking (Britton et al., 2004; Shipton et al., 2009). Given the effects of smoking on the developing fetus and the high costs of many medical procedures, the economic and moral imperatives to improve detection and treatment of smoking are considerable.

In response to this need for identifying active smokers, many clinicians elect to use either exhaled carbon monoxide (CO) or cotinine assessments. Exhaled CO levels are easy to perform. Unfortunately, CO has a short half-life (Florescu et al., 2009). As a result, these tests are insensitive to intermittent smoking and individuals can easily avoid detection by simply not smoking in the hours before a clinic appointment (Florescu et al., 2009; Cropsey et al., 2014). Assessments of cotinine, a metabolite of nicotine, which can be performed on serum, urine, or hair samples, are generally regarded as the most reliable biomarker for the screening of smoking (Florescu et al., 2009). Unfortunately, nicotine replacement therapies, which are used by up to 90% of smokers undergoing smoking cessation therapy, also results in the generation of cotinine and substantial numbers of ex-smokers report being unable to discontinue nicotine replacement without resumption of smoking. Hence, all individuals need to do to evade detection is to claim that either they are “vaping” (i.e., using e-cigarettes) or using nicotine replacement therapy. Conceivably, these false reporters could be detected by the quantification of pyrholized tobacco products (Jacob et al., 2002; Florescu et al., 2009). But currently, the approaches to detect these oxidized tobacco byproducts require complex and expensive procedures.

Methylation sensitive droplet digital PCR methods may offer a more scalable, sensitive, and specific approach to assessing cigarette consumption. In 2012, we reported that DNA methylation status at cg05575921, a CpG locus in the aryl hydrocarbon receptor repressor (AHRR) was significantly associated with smoking status (Monick et al., 2012). Since that time, over 50 studies using these genome wide tools have replicated this finding (for a partial listing, please see Andersen et al., 2015; Gao et al., 2015) with other studies extending the relationship of methylation of this locus to key healthcare outcomes including mortality, cardiac risk, and lung cancer (Zhang et al., 2016; Bojesen et al., 2017).

Although stimulating, due to their high cost, complexity of data processing and long turnaround time, these array based approaches for measuring cg05575921 methylation are not clinically implementable as smoking assessment tools. Therefore, in 2015, we developed a methylation sensitive quantitative PCR method for assessing DNA methylation at cg05575921 (Dogan et al., 2014). However, although qPCR can be rapidly and cheaply performed, due in part to their need for external reference standards, qPCR approaches have significant limitations in their precision and accuracy (Hayden et al., 2013). The recent introduction of digital PCR methods largely circumvents many if not most of these difficulties while providing rigorous confidence interval estimates for methylation measurements (Hayden et al., 2013; Dogan et al., 2014; Maheshwari et al., 2017).

In this communication, we describe the droplet digital implementation of a ddPCR assay for cg05575921 methylation and its relationship to Illumina array measurements. Then, using data from subjects specifically collected for quantitative substance consumption analyses, we determine sensitivity and specificity of this assay for detecting smoking in adults and the relationship of cg05575921 demethylation to cigarette consumption across the life cycle.

## Materials and methods

The current study uses clinical data and biomaterials from subjects collected from two NIH funded protocols both of which were approved by the Western Institutional Review Board (www.wirb.com), WIRB approval 20160135 and 20162083.

### Clinical characterization of subjects

The first protocol ascertained heavy drinking subjects and alcohol abstinent controls (Philibert et al., in press). The heavy alcohol consuming subjects were recruited from one of three Iowa substance use treatment organizations; Center for Alcohol and Drug Services (CADS, Davenport, IA), Prelude Behavioral Services (campuses in Iowa City and Des Moines, IA) and Alcohol and Drug Dependency Services of Southeast Iowa (ADDS, Burlington, IA) using our previously described protocol (Philibert et al., 2014). In brief, between 1 and 7 days after their last intake of alcohol, adults who were admitted for detoxification were referred for participation in the study by staff or via printed posters concerning the study. These individuals were not considered for inclusion if under the influence of any substance. After full informed written consent was received, each subject was interviewed with a series of instruments including a modified form of Version II of the Semi Structured Assessment for Genetic Studies (SSAGA-II) and our Substance Use Questionnaire, an inventory that assesses substance consumption over recent time periods (Selzer, 1971; Bucholz et al., 1994; Philibert et al., 2014). After interview, the subjects were phlebotomized to provide DNA and sera for the study. Serum and DNA were prepared and stored as previously described (Philibert et al., 2014). Only subjects who reported current smoking were considered for inclusion in this study.

Non-smoking control subjects for this first protocol were collected at the University of Iowa site using our previously described protocol (Philibert et al., 2014). In brief, control subjects who denied consumption of alcohol in the past year and a lifetime history of any substance use disorder, with the exception of tobacco use disorder were solicited for the inclusion in the control arm of the overall study. After consent was received, the subjects were interviewed with the SSAGA and the Substance Use Questionnaire, and then phlebotomized to provide biomaterial for our studies. Importantly, only those subjects who denied significant lifetime use of smoking either tobacco (<100 cigarettes) or tetrahydrocannabinol (THC) (<21 times) were considered for the sensitivity and specificity analyses described in this study.

A second cohort of smoking subjects were collected as part of an observational study of smoking cessation subjects at the Davenport CADS site. In brief, before consideration for inclusion, subjects 18 years or older were prescreened to ensure interest in smoking cessation, current cigarette consumption at least 8 cigarettes per day, 5 pack years of cigarette consumption and an exhaled CO of ≥ 8 ppm. Subjects meeting those criteria were referred for medication management, then offered inclusion in an observational study of smoking cessation. After consent, each of these subjects were also interviewed with the SSAGA and Substance Use Questionnaire, then phlebotomized to provide biomaterials for this study.

### Methylation methods

The array measurements of cg05575921 methylation were extracted from an Infinium MethylationEpic Beadchip data set (GSE 110043) obtained from a recently conducted genome wide analysis of the relationship of DNA methylation to alcohol consumption status (Philibert et al., in press). The University of Minnesota Genomics Center conducted these measurements using reagents and protocols from the manufacturer (Illumina, San Diego, USA) (Pidsley et al., 2016). After the methylation intensity data (IDAT) files were downloaded from their secure server, we conducted probe filtering, background correction and adjustment for probe types using the MethyLumi, WateRmelon and FDb.InfiniumMethylation.hg19 R packages (Pidsley et al., 2013; Triche, 2014; Davis et al., 2017). Quality control was performed on the sample and probe levels with all values for cg05575921 methylation surviving quality control.

The methylation status of cg05575921 was determined for each of these samples using a droplet digital PCR paradigm featuring a Bio-Rad (Carlsbad, CA) QX-200 Droplet Reader and an Automated Droplet Generator (AutoDG) In brief, 1 μg of DNA from each subject was bisulfite converted using the Fast 96 Bisulfite Conversion kit (Qiagen, Germany). An aliquot of each sample was pre-amplified, diluted 1:3,000, and then PCR amplified using fluorescent, dual labeled primer probe sets specific for cg05575921 from Behavioral Diagnostics (Coralville, IA, USA; available via sale via IBI Scientific, Dubuque, IA, www.ibisci.com) and Universal Digital PCR reagents and protocols from Bio-Rad (Carlsbad, CA, USA). The number of droplets containing amplicons that have at least one “C” allele (corresponding to the methylated cytosine residue), at least one “T” allele, at least one “C” and “T” allele, or no amplifiable alleles was determined using a QX-200 droplet counter and QuantiSoft software lasso function (Bio-Rad, USA). The results were expressed as a percent methylation (Andersen et al., 2017). Reactions were excluded if fewer than 10,000 droplets were counted or the 95% confidence interval for the mean of the observed value exceeded 3%.

### ELISA methods

Serum cotinine and tetrahydrocannabinol (THC) levels were obtained for all subjects using quantitative cotinine and THC enzyme linked immunoassays (ELISA) kits from AbNova (Taiwan) according to the manufacturer's directions. Absorbance of each sample was determined using a Molecular Devices (Sunnydale, USA) EMax spectrophotometer.

### Statistical methods

To demonstrate the predictive capability of smoking status using the ddPCR assay, data from all 177 subjects (98 smokers and 78 controls) were randomly split into training (60%) and testing datasets (40%). The training and testing datasets consisted of 107 (60 smokers and 47 non-smokers) and 70 subjects (39 smokers and 31 non-smokers), respectively. A binary logistic regression model (100 bootstrap repetitions) was fitted in R using training set data to predict the probability of being a smoker using DNA methylation at cg05575921. A false negative misclassification cost twice as much as a false positive misclassification cost, was set to determine the prediction probability cutoff. The area under the curve (AUC) of the Receiver Operator Characteristic curve, sensitivity, and specificity of each bootstrap repetition were calculated. To evaluate the performance of the model on the test set, logistic regression was performed on the training data and was saved for testing on the test set. This approach was repeated to include age and gender in the prediction model.

Other non-genome wide quantitative analyses of both array and ddPCR derived methylation data were conducted using JMP Version 10 software (SAS, Cary, NC USA) and the specific routines are outlined in the text.

## Results

### Subject characteristics

The clinical and demographic variables of the smoking and non-smoking cohorts are given in Table 1. Overall, the average age of the controls was 6 years greater than that of the smokers (*p* < 0.001). In addition, the largely White controls were more likely to be female (62%) while the smoking subjects were more likely to be male (66%). The smoking subjects who were recruited from the alcohol consumption study tended to be heavier smokers than the subjects recruited from the smoking cessation study (*P* < 0.01 at 1 month, 6 months, and 1 year consumption windows). However, their self-reported life time consumption (in pack years) of the two groups was very similar.

**Table 1:** Key clinical and demographic data on study subjects.

	**Controls**	**Smoking cessation**	**Alcohol consumption**
N	78	36	63
Age	46.7 ± 14.5	39.7 ± 12.5	40.4 ± 10.5
Ethnicity			
White	70	31	52
African American	2	2	8
Other	6	3	3
Gender			
Male	32	21	44
Female	46	15	19
Cigarettes per day			
Past Month	–	11.9 ± 6.7	18.8 ± 12.0
Past 6 Months	–	14.8 ± 7.6	19.7 ± 13.1
Past Year	–	14.7 ± 8.4	19.7 ± 13.1
Life Consumption Pack Yr		15.5 ± 8.4	14.5 + 12.0
Average cg05575921 (%)	85.9 ± 3.6	50.4 ± 13.9	47.2 ± 17.1
FTND Score	–	4.3 ± 1.6	5.0 ± 1.9
Some Cannabis Past Year?	0	19	26

*“±” indicates the standard deviation of the sample*.

### Methylation sensitive ddPCR assessments of DNA samples

**Figure 1:**
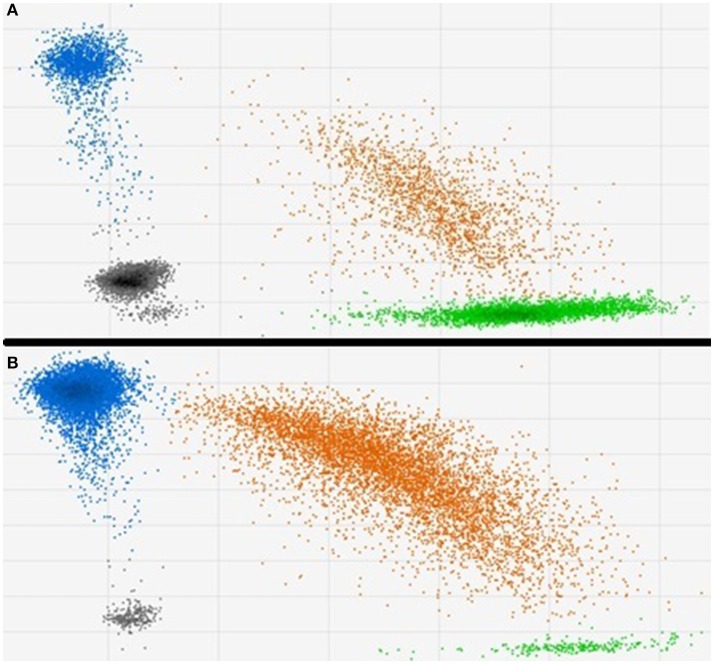
Typical 2D plots of droplet counts by the fluorescent droplet counter. **(A)** Shows the results from a 19 cigarette per day smoker. Methylation at cg05575921 was 29.0% with 15,910 total events, 3,607 “at least one blue” channel (Fam label) + events and 7,441 “at least one green” (Hex) + events have been observed. **(B)** Shows the results from a non-smoker. Methylation was 86.4% with 15,824 total events, 15,291 “at least one blue” channel (Fam label) + events and 6,560 “at least one green” (Hex) + events have been observed.

The results from two typical methylation sensitive ddPCR assessments of cg05575921 methylation in DNA from a smoker and non-smoker are shown in Figure 1. In essence, treatment of DNA with sodium bisulfite transforms a potential methylation difference into a potential quantitative genotype difference. After exposure to bisulfite, methylated cytosines remain unconverted while the unmethylated cytosines are converted to uracils which are subsequently read by DNA polymerases as thymines. In the next step of the ddPCR process, the bisulfite converted amplicons are pre-amplified under high stringency conditions. Then, approximately 20,000 of those amplicons are added to a 22 μl PCR mixture that contains allele specific fluorescent hydrolysable probes. The resulting mixture is then mechanically partitioned into ~20,000 individual oil encapsulated droplets for PCR amplification. After PCR, the droplets are pulled through a flow cytometer which fluorescently interrogates each droplet to discern the degree of probe hydrolysis with respect to each allele. The difference of methylation between smokers and non-smokers as quantified by this process is visibly evident in the 2D displays of the two samples. In the DNA from the non-smoker, the cg05575921 cytosine residue is heavily methylated (86%) with the vast majority of droplets are interpreted as having had all “C” alleles (shown as blue) or one or more C and T alleles, shown as orange (Figure 1B). In contrast, in results obtained using DNA from a pack a day smoker (Figure 1A), the vast majority of the droplets have one “T” alleles (shown as green) or one or more C and T alleles, shown as orange which reflect the demethylated status of cg05575921 (29%).

**Figure 2:**
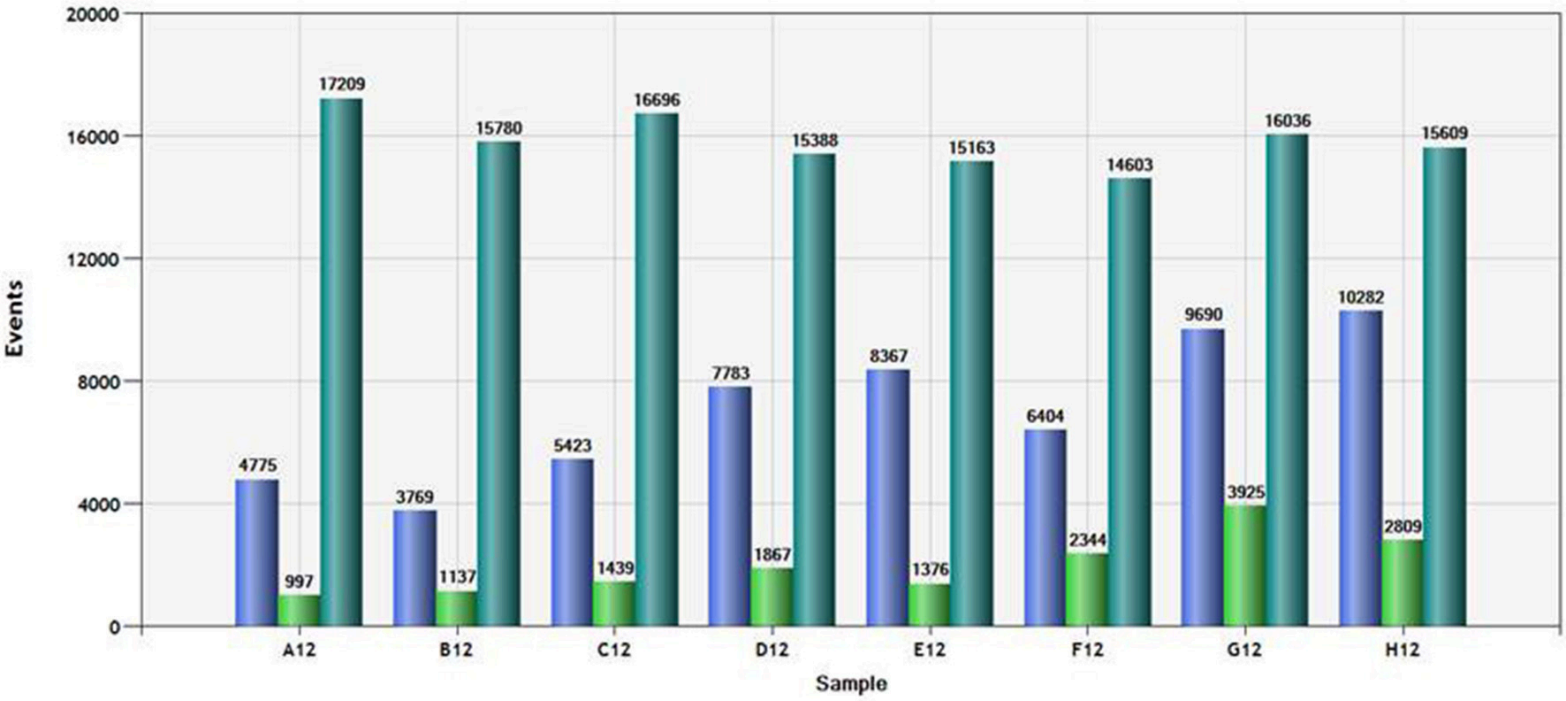
Total count and channel specific droplet count for a typical column of samples. Droplets positive for at least one “T allele” (green bar), positive for at least one “C allele” (“blue bar” and total number of droplets (positive and negative) are shown for each of the samples in the plate column.

Since the QuantiSoft software imputes percent methylation using a Poisson distribution function, adequate number of counts of each allele are necessary to ensure precision. Figure 2 shows the total number of droplets observed and the counts of each channel (“green” or “blue”) for a typical column of samples. A total of 15,000 counts for each sample was generated then read by the Bio-Rad ddPCR system. The total number of droplets that contained at least one “C allele” (Blue) ranged from 3,769 to 10,282. The total number of droplets that contained at least one “T allele” (Green) ranged from 997 to 3,925. In our experience using this assay, counting fewer than 4,000 total positive events (either blue or green) is associated with steadily increasing confidence intervals for the observed mean methylation.

The cg05575921 methylation in the controls ranged from 72.5 to 90.1% with an average of 86%. In contrast, the range of cg05575921 methylation in the smoking subjects ranged from 19.5 to 83.5% with an average methylation of 48.4%.

**Figure 3:**
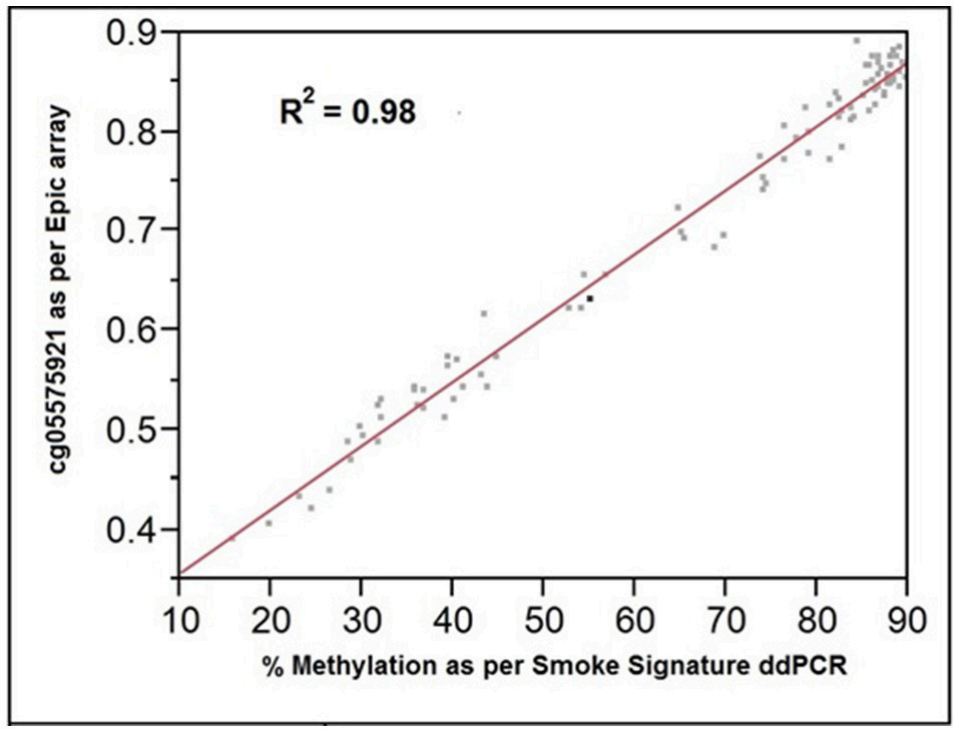
The relationship of cg05575921 methylation as assessed by the Illumina Epic array (expressed as fractional methylation) and the Smoke Signature ddPCR assay (expressed as % methylation). *R*^2^ = 0.98, *n* = 92.

The comparability of the ddPCR and the Illumina array assessments of cg05575921 methylation is shown in Figure 3. The assessments were highly correlated (*R*^2^ = 0.98; Pearson). The standard error of ddPCR assessments of independently prepared replicate samples was 0.7% with no significant batch effect.

### Biochemical verification of self report of substance consumption

Our prior experience has demonstrated that subjects recruited for our behavioral studies may misrepresent their substance use status. To minimize the possible effect of misrepresentation, the accuracy of ddPCR determination of cg05575921 status to classify smoking status was examined using only subjects whose smoking status was biochemically verified. To do this, we conducted ELISA testing on sera from self-professed daily smokers and cotinine negative controls who denied a history of substantial tobacco smoking (>100 cigs lifetime), a substantial lifetime use of THC (more than 21 uses), and no use of either substance in the past year. The biochemical status of all smoking subjects matched their self-reported status. However, despite explicit questioning, serum testing of 6 of the 84 potential control subjects showed the presence of cannabinoids or above background cotinine (>1 ng/ml) inconsistent with their self-reported non-smoking status. The data from these six subjects was excluded from further analysis or inclusion in Table 1.

### Sensitivity and specificity analyses

To determine whether cg05575921 methylation status in the biochemically confirmed subjects predicts smoking status, we conducted logistic regression (100 bootstrap repetitions) of cg05575921 methylation to smoking status (smoker vs. non-smoker) alone and with age and gender on the training set. For the 100 repetitions, the range of ROC AUC, sensitivity and specificity were 0.927–1.000 (mean = 0.994, std = 0.011), 0.760–1.000 (mean = 0.967, std = 0.054) and 0.769–1.000 (mean = 0.924, std = 0.054), respectively. A final logistic regression model was then fitted to the training data. The intercept and methylation coefficients were 36.2675 and −0.4750, respectively. The probability cutoff was determined to be 0.2384 (corresponding to a cg05575921 methylation value of 78.8%). Table 2 shows the performance of this final model on the test data. The test ROC AUC, sensitivity and specificity are 0.993, 0.897 and 0.968.

**Table 2:** Confusion matrix for test set.

**cg05575921 Only model**			**cg05575921, Sex and age model**		
***TRUE***	**Smoker**	**Non-smoker**	***TRUE***	**Smoker**	**Non-smoker**
**TEST SET PREDICTED (*n* = 70)**					
Smoker	35	4	Smoker	37	2
Non-smoker	1	30	Non-smoker	1	30

**Figure 4:**
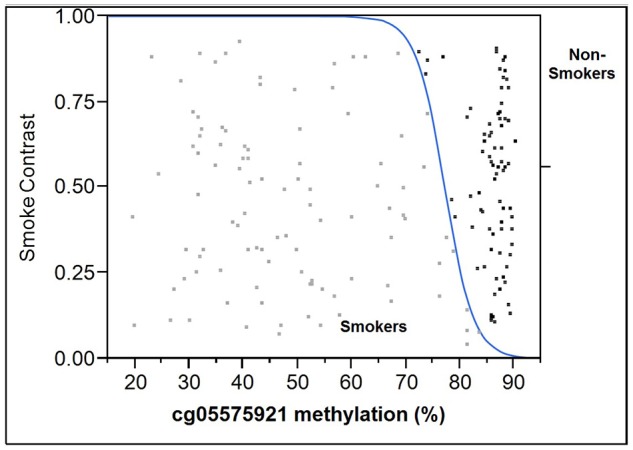
Logistic plot of the relationship of cg05575921 methylation to smoking status. The results from the smokers (*n* = 99) are in light gray and are to the left of the blue curve while the results from the non-smoking subjects (*n* = 78) are black and are to the right of the blue curve.

When age and gender were included, for the 100 repetitions, the range of ROC AUC, sensitivity and specificity were 0.845–1.000 (mean = 0.977, std = 0.034), 0.750–1.000 (mean = 0.939, std = 0.062) and 0.737–1.000 (mean = 0.947, std = 0.059), respectively. A final logistic regression model was then fitted to the training data. The intercept, methylation, age and gender coefficients were 45.6683, −0.5481, −0.0925 and −0.1411, respectively. The probability cutoff was determined to be 0.1183. Table 2 shows the performance of this final model on the test data. The test ROC AUC, sensitivity and specificity are 0.994, 0.949 and 0.968. In order to better visualize the relationship between cg05575921 status and smoking status, a logistic regression plot using the data from all 177 subjects simultaneously is given in Figure 4.

### Examining the dose dependency of methylation

Next, using the data from the SSAGA and our Substance Use Questionnaire (SUQ), we examined the relationship of AHRR methylation to cigarette consumption. The SUQ specifically quantifies average consumption of each type of tobacco, including cigarettes, cigars, and chew, over the past 1 month, 6 months, and 1 year thresholds, while the SSAGA is useful for obtaining lifetime pack year cigarette consumption estimates. As an initial step of our analyses, we determined the relationship of the consumption variables to one another. Not surprisingly, each of the SUQ variables were highly correlated to each other (for each comparison, *R*^2^ > 0.82) with lesser, yet still significant, correlation of each of them to lifetime consumption index (for each comparison *R*^2^ < 0.09 with all *p* < 0.02). Using the consumption estimates from these inventories, we next analyzed the relationship of cg05575921 methylation, as assessed by ddPCR, to each of those variables. Overall, methylation status was best correlated with average cigarette consumption over the past year (*R*^2^ = 0.47; *p* < 0.0001), with lesser correlations for average 6 month, 1 month, and lifetime consumption indices (*R*^2^ values of 0.42, 0.46, and 0.04, respectively). A linear model of methylation to average consumption over the past year showed that every 1% decrease in methylation corresponded to a 1.2 cigarettes per day.

Finally, we analyzed the relationship of cigarette craving, as tabulated by the Fagerstrom Test for Nicotine Dependence (FTND) and Carbon Monoxide levels, to self-reported and methylation indices of cigarette consumption. FTND score significantly predicted average cigarette consumption over the past year (*p* < 0.05; *R*^2^ = 0.04; *n* = 97) but not cg05575921 methylation.

## Discussion

Establishing a “normal range” of cg05575921 methylation for non-smokers is not a trivial task. In fact, a recurrent major challenge for biomarker development for all types of behavioral illnesses is the identification of “clean controls.” Our current study recruited control subjects from a university employee population with specific emphasis on recruiting individuals with religious prohibitions against substance use. Still, 7% of potential control subjects who denied any tobacco or cannabis use over the past year had ELISA results indicative of recent use. This finding is consistent with the results from our prior attempts to identify “clean controls” for substance use studies (Philibert et al., 2014). Because the ELISAs used in herein detect only recent usage, it is quite possible that many of the “clean” subjects, in particular those with lower AHRR values, had substantial prior histories of tobacco or cannabis use. But that is speculation. To better establish the “non-smoking” range for DNA methylation, we are following a cohort of 450 Iowa high school sophomores using a repeated sampling strategy to detect surreptitious substance use under the suppositions that most 16 year-old subjects, if they smoke, are only in the experimental phase of smoking, and that those who are at least intermittent smokers, will either self-report use or turn up ELISA positive in the two follow waves of bio-sampling. Indeed, the results to date from that Iowa study and the results from our 2015 cross sectional analysis of 16 year old subjects from Georgia very much support a cg05575921 normal range whose corresponding ddPCR derived methylation value is above 82% (Philibert et al., 2012). However, collection from our more rigorous longitudinal study will not be completed for another year. So, for the time being, after considering the cutoff rate empirically derived in our training set (81.4%) and eye-balling the overall plot shown in Figure 4, we suggest that the normal range for non-smokers for this assay is from 82% and above, with all other values, particularly those below 75% be considered as being from those individual with at least a lifetime, if not current, history of smoking.

A frequent question that we are asked is “*what is the effect of smoking cannabis on the test*.” In our experience of examining thousands of subjects with this assay, the answer is clear, repeated cannabis smoking results in demethylation of AHRR. Pyrolysis of cannabis, like tobacco, results in the generation of polyaromatic hydrocarbons (PAH) whose inhalation will lead to activation of AHRR (Ding et al., 2005; Moir et al., 2008). But determining the exact effects of smoking cannabis on cg05575921 methylation may be difficult or impossible for a number of reasons. First, in order to increase profits or improve the burning characteristics of cannabis, tobacco is often mixed with the marijuana (Peters et al., 2012). Second, between 25 and 52% of all tobacco smokers also use cannabis periodically, while between 41 and 94% of all cannabis users smoke cigarettes which makes recruiting “pure” cannabis or tobacco consumers difficult (Peters et al., 2012). Third, the amount of PAH ingested by the various methods of smoking cannabis is poorly constrained. Hence, for a given amount of THC consumption, the amount of PAH exposure between cannabis consumers may greatly vary. Finally, in our direct experience, self-report of cannabis use by substance users is very unreliable. For example, while screening potential samples for inclusion in our initial case and control study of smoking associated DNA methylation in middle aged African American adults (MH080898), 80% of those samples with ELISA values strongly indicative of recent cannabis use came from subjects who denied lifetime use of cannabis (Monick et al., 2012). Hence, given the societal biases against cannabis consumption and the need of biomarker studies to have stringently defined cases and controls, we believe it will be difficult to provide a rigorous understanding of the relationship of cannabis consumption to AHRR methylation in the near future.

A second question that is frequently asked is “*how quickly does the methylation signature at AHRR revert to normal?”* In particular, this question is of critical importance to those of us who wish to use changes in methylation to guide tobacco cessation therapy. AHRR methylation clearly reverts as a function of smoking cessation (Tsaprouni et al., 2014; Bauer et al., 2016; Philibert et al., 2016; Wilson et al., 2017). However, the exact shape of the reversion curve is poorly constrained for a number of reasons. First, the majority of smokers who manage to quit smoking relapse before achieving final cessation (de Jesus et al., 2016). Hence, longitudinal studies of smoking cessation will suffer a steady attrition of non-smoking subjects. Second, in our experience, smokers rarely stop “cold turkey.” Instead, in our two commercial studies of smoking cessation where we biochemically checked smoking status monthly (CA213507; DA037620), none of the subjects quit “cold turkey.” Instead, each of the smokers who managed to quit tapered down cigarette consumption over a period of weeks to months. Finally, as evidenced in Table 1, those contemplating smoking cessation treatment often reduce cigarette consumption before presenting for treatment. As a result of these other phenomenological characteristics, from a methodological standpoint, it may be difficult to get a blood samples representing cleaning defined periods of consumption from the time before cessation through the necessary future time periods to that are necessary to more exactly determine the reversion curve of AHRR methylation in response to smoking cessation.

The finding that the current AHRR methylation is better related to the average daily consumption over the past year rather than the past month will be of use in conceptualizing the “real life” tempo of environmental induced epigenetic reprogramming. On the shortest time scales, changes in gene expression are moderated through transcriptional co-activators and repressors (Voss and Hager, 2013; Bintu et al., 2016). However, on longer timescales, epigenetic changes are thought to play a larger regulatory role (Bintu et al., 2016). Some of these epigenetic changes, such as histone modifications, can occur within minutes with changes in DNA methylation being thought to occur over the course of hours to days. However, the timing of most of these epigenetic changes has been determined using *in vitro* paradigms and non-physiologically relevant environmental exposures. Whereas clearly informative, these *in vitro* experiments cannot take into account *in vivo* factors such as the effects of tar deposition and the chronic inflammatory state that occurs as a consequence of smoking (Sinden and Stockley, 2010). The current findings that show that average consumption over the past 1 month (*R*^2^ = 0.46) and past 6 months (*R*^2^ = 0.46) were the best predictors of AHRR methylation lend modest support to the suggestion that the time scale for changes in this portion of the methylome with alterations of cigarette consumption is on the weeks or months. Studies of methylation changes secondary to alteration of other substances such as alcohol may contribute additional information on the response characteristics of the peripheral white blood cell methylome to beneficent interventions. However, we will note that although not yet well defined, the time scale for the reversion of changes of the methylome in heavy alcohol consumers suggest the period of abstinence required to achieve 50% reversion of the alcohol induced changes is on the order of months as well (Philibert et al., 2014).

We did not find any relationship of cg05575921 methylation to FTND. Given the rather modest relationship of cigarette consumption to FTND score in our subjects, this might simply be an issue of insufficient power. Still, it is important to realize that nicotine is the major addictive substance in cigarettes and whereas AHRR is a potent feedback modulator of Cyp1A1 and Cyp1A2, it does not regulate nicotine metabolism which instead involves Cyp2A6 (Hukkanen et al., 2005; Nguyen and Bradfield, 2007). Indeed, the use of “chew” has no effect on cg05575921 methylation (Besingi and Johansson, 2013; Philibert et al., 2015). Still, there are other potentially neuroactive substances such as carbon monoxide (CO) in tobacco smoke and CO may modulate cigarette craving (Milne et al., 2012; Hanafy et al., 2013). Since both PAH and CO are generated through pyrolysis, we are engaging in additional studies to determine whether there is a relationship between CO levels and cg05575921 levels, and whether a combination of nicotine and cg05575921 levels better capture craving than either measure alone.

In conclusion, we report that ddPCR assessments of cg05575921 methylation can be used to accurately determine smoking status in adults and lay the groundwork for a better understanding of the dose response relationship of cigarette smoking to demethylation at this locus. Further studies to determine the dose response relationships in adolescents and the reversion curve of methylation in former smokers are indicated.

## Author contributions

RP obtained funding, conducted some of the analyses and wrote the initial draft of the manuscript. MD conducted the machine learning analyses and edited the manuscript. AN, BK, SM, and EP collected and characterized the subjects in the study and edited the manuscript. JL provided commentary to the manuscript and analytical support to MD. SB and DB edited the manuscript. JC and DB also supervised the collection of the subjects.

### Conflict of interest statement

RP is the Chief Executive Officer of Behavioral Diagnostics and inventor on a number of granted and pending patent applications with respect to both alcohol and tobacco consumption related to the material discussed herein. The use of cg05575921 status to determine smoking status is protected by US Patents 8,637,652 and 9,273,358. The other authors declare that the research was conducted in the absence of any commercial or financial relationships that could be construed as a potential conflict of interest.
